# Patient-Specific Numerical Simulations of Endovascular Procedures in Complex Aortic Pathologies: Review and Clinical Perspectives

**DOI:** 10.3390/jcm12030766

**Published:** 2023-01-18

**Authors:** Lucie Derycke, Stephane Avril, Antoine Millon

**Affiliations:** 1 Department of Cardio-Vascular and Vascular Surgery, Hôpital Européen Georges Pompidou, F-75015 Paris, France; 2 Centre CIS, Mines Saint-Etienne, Université Jean Monnet Saint-Etienne, INSERM, SAINBIOSE U1059, F-42023 Saint-Etienne, France; 3 Department of Vascular and Endovascular Surgery, Hospices Civils de Lyon, Louis Pradel University Hospital, F-69500 Bron, France

**Keywords:** finite element, endovascular aortic repair, numerical simulation, computational modeling

## Abstract

The endovascular technique is used in the first line treatment in many complex aortic pathologies. Its clinical outcome is mostly determined by the appropriate selection of a stent-graft for a specific patient and the operator’s experience. New tools are still needed to assist practitioners with decision making before and during procedures. For this purpose, numerical simulation enables the digital reproduction of an endovascular intervention with various degrees of accuracy. In this review, we introduce the basic principles and discuss the current literature regarding the use of numerical simulation for endovascular management of complex aortic diseases. Further, we give the future direction of everyday clinical applications, showing that numerical simulation is about to revolutionize how we plan and carry out endovascular interventions.

## 1. Introduction

Personalized medicine involves managing health based on individual characteristics and has great potential to improve the care of patients suffering from cardiovascular diseases [1,2]. In recent years, research in this field has focused significantly on gene-targeted drug therapy. Meanwhile, trans-catheter implantable devices, such as coronary and peripheral stents, aortic valves, and stent-grafts (SG), represent a continuously increasing part of cardiovascular therapeutic armamentarium. Despite numerous design improvements and the growing expertise of interventionists, precise prediction of device behavior in a particular patient anatomy remains a challenge, particularly in complex aortic disorders. Large inner- and intra-individual variability exists, implying uncertainty in the patient-specific geometry, mechanical properties, and biological function of the aorta. Therefore, in complex aortic pathologies, endovascular procedures require advanced skills and experience, with associated significant learning curves [3,4]. Moreover, currently used tools for procedure planning are mostly limited to computed-tomography (CT) scan image reconstruction software. One of the main limitations of such tools is the inability to provide personalized prediction of device behavior.

Numerical modeling is a digital technique used to numerically reproduce various biomechanical behaviors. Unlike open surgery, endovascular aortic repair is essentially based on the biomechanical interactions between an SG and the aortic wall. Material properties of the aortic wall, stents, and fabrics are now mostly identified [5,6,7,8,9,10,11,12,13,14,15]. The use of numerical modeling in endovascular aortic surgery, particularly in the field of mechanical interaction analysis, has the potential to reduce uncertainties by predicting aortic device behavior in a personalized aortic anatomy. More tailored treatment could be obtained. Although computational models are still a relatively new development in this area, advancement is occurring at a rapid rate. In this review, we present the principles of numerical modeling with the steps that are generally followed in simulations and address the most relevant efforts at using numerical simulation in the endovascular management of complex aortic pathologies. We also present a focus on recently reported applications and discuss the unresolved limits and future directions of this emerging technology.

## 2. Methodology of Numerical Simulations

**Figure 1:**
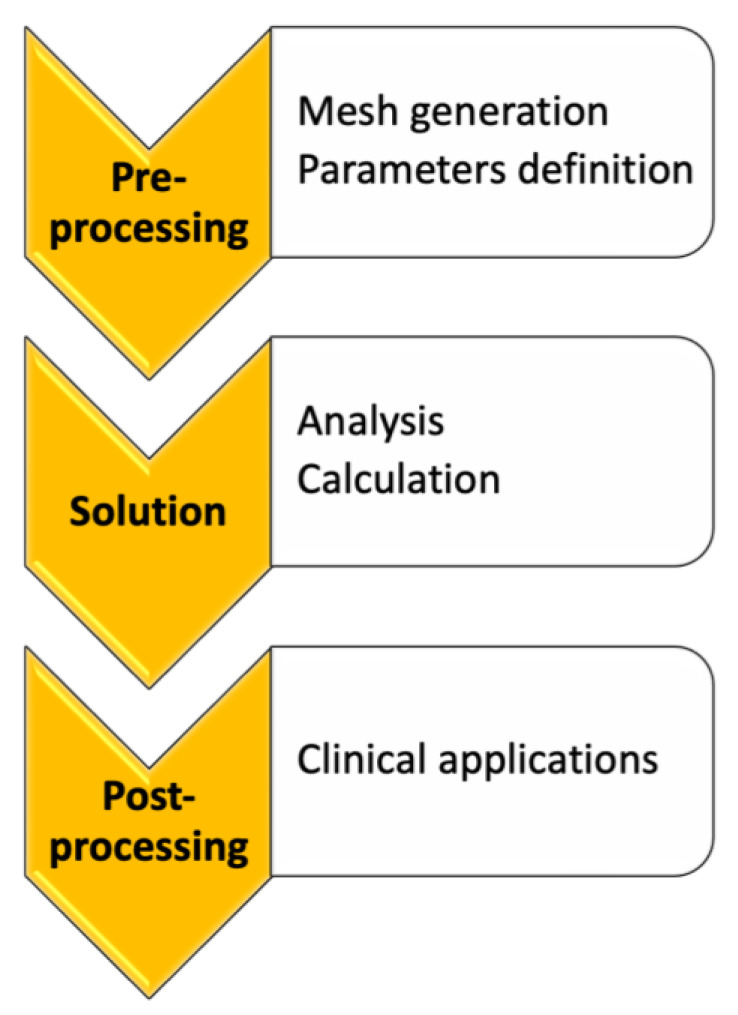
Schematic representation of simulation methodology.

The approach of numerical modeling is aimed at assessing variations in space and time of various components (e.g., SG behavior, blood velocity, or wall shear stress). This involves solving a set of differential equations and modeling physical phenomena (e.g., blood fluid dynamics) in a specific discretized domain, called a mesh (e.g., an aortic-shaped geometry). The process consists of three main steps of simulation: (1) preprocessing, (2) solution with different types of analysis and calculation methods, and (3) post-processing, as illustrated in Figure 1.

### 2.1. Preprocessing: From Medical Images to Patient-Specific Model

The first step of a computer simulation is segmentation from vascular imaging and discretization or meshing. This means that volumes of the segmented lumen and wall are divided into small triangles or quads [16]. A finer mesh gives more accurate results but requires more computational resources and time. A common practice is to find the best tradeoff between these two criteria. Currently, many dedicated tools, some of which are open-source, allow semi-automatic segmentation (meaning with only a few manual selections of targets points) of CT or MRI images and creation of 3D vascular models [17,18,19,20]. Subsequently, these models can be used for specific analysis purposes. Various degrees of accuracy exist, depending on whether or not different parameters are taken into account, such as intra-luminal thrombus or arterial wall calcification.

Material properties are then numerically assigned to each element of the mesh. Therefore, meshes not only represent the geometry, but also contains physical information. Boundary conditions are defined, which refer, for instance, to how the structure is attached and/or interacts with its environment, such as aortic contacts with the spine and surrounding organs [16].

Additional data can be added from medical images and can be used to define boundary conditions or material properties. For example, inlet/outlet blood velocity profiles with four-dimensional phase contrast MRI and composition of atherosclerotic plaques, including lipid core, calcifications, or fibrous cap, can be extracted from MRI [21,22].

### 2.2. Solution

#### 2.2.1. Types of Analysis

In a numerical simulation, analysis consists of solving equations that represent the physics of the phenomena of interest. These phenomena can include the motion of a fluid (computer fluid dynamics), deformation of a solid (computational structural dynamics), or a combination of both (fluid-structure interactions). The choice of method depends on the type of problem studied.

- Computational Structural Dynamics

Structural mechanics analysis is a numerical method for solving mechanical problems in a biological environment. Structures can be numerically reproduced geometrically and assigned with mechanical properties. Therefore, such structures can behave mechanically like an SG or aortic wall. In the cardiovascular area, this numerical simulation has the potential to predict trans-catheter implantable device deployment, such as aortic valves and aortic SG [14,23,24,25,26,27,28].

- Computational Fluid Dynamics (CFD)

Fluid dynamics analysis is usually adopted in cardiovascular biomechanics for the assessment of hemodynamic conditions of a vessel segment [29]. The vessel wall is basically assumed to be rigid, neglecting the interaction between the arterial wall and pulsatile blood pressure. This method is very useful in many applications where the impact of wall deformations on hemodynamics can be neglected. A major determinant of such analyses are the boundary conditions, i.e., the flow/pressure conditions at the inlets and outlets [30,31]. Among hemodynamic factors, wall shear stress (WSS), i.e., the viscous shear applied to the endothelial cells, has been studied the most [29].

- Fluid-Structure Interaction (FSI)

As the blood and vessel wall always interact mechanically, it is sometimes needed to resort to FSI analyses. This method involves solving the problem of interaction between a deformable solid and fluid [29]. This can become crucial, for example, for simulating the motion of the intimal flap in a dissection or the effect of aortic valve regurgitation. However, given their high complexity, FSI analyses require very long calculation times, which are usually not compatible with clinical practice.

#### 2.2.2. Calculation Methods

Many computational methods for solving the equations have been developed. Here, we present the most known and used methods.

- Finite Element (FE) Analysis

FE analysis is a numerical method for solving equations after discretizing the domain of interest into a large number of small elements, called finite elements. This method represents approximately 90% of the numerical methods used for performing the analyses described above. FE simulation studies performed on percutaneous valves [23,24] and simple SG designs for aneurysms [14,32,33] have shown the significant potential of this technology for cardiovascular medicine. More complex and relevant models were then developed, their review is the purpose of our present work.

- Machine Learning (ML)

ML includes data-science tools that use neural networks to learn the solution of a problem from thousands of existing solutions by analyzing the relationship between input and output data and detecting meaningful patterns. These existing solutions can be measured or simulated by FE analysis. Current ML efforts in the area of aortic diseases focus on improving image segmentation and characterizing aneurysm morphology, geometry, and fluid dynamics [34]. For instance, the maximal aneurysm aortic diameter is measured manually via post-operative CT scan to monitor and detect complications. This measurement is currently still affected by intra- and inter-observer variability and is time-consuming. Maximum aortic diameter measurements using a fully automated solution developed by an artificial intelligence start-up company (Incepto^®^) were recently assessed in a cohort of patients (489 cases for training and 62 for validation) and compared with those of seven clinicians, showing good accuracy [35].

Once trained, ML is very fast. It can therefore be used in digital twins, which require a real-time response. Indeed, the digital twin, defined as a virtual representation of its living counterpart, the physical twin, requires dynamic updating of data to maintain a relevant representation of the physical twin. Recently, Chakshu et al., described a method for detecting abdominal aortic aneurysm and its severity in a virtual cohort of patients based on analyzing three blood pressure waveforms [36]. However, although this method represents a promising solution to the screening issue, the technology used idealized vessel wall properties and morphological geometries and did not consider calcified or thrombus regions, leading to many biases.

The technology is currently still in its infancy but improved computer capabilities and dataset collection in the near future will probably extend its impact in the field of complex aortic diseases.

### 2.3. Post Processing: From Numerical Outputs to Clinical Practice in Endovascular Aortic Repair

The development of FE simulations to assist cardiovascular interventions started a decade ago [5,13,37]. SG mechanical properties were characterized using bench tests and used to achieve FE analyses of SG deployment in virtual aortas and iliac arteries. The term “virtual model” refers to an idealized model that is not created from patient anatomy. It differs from the “patient-specific model” which reproduces the geometry of a given patient’s anatomy. These models highlighted the importance of boundary conditions, contacts, and material properties [5,6,7,25,26]. Moreover, they established the basic assumptions about pre-stressed conditions, arterial wall properties, and interactions with surrounding tissues [15,38,39]. Recently, more complex numerical simulations of bifurcated and fenestrated SG deployment in patient-specific models have shown the validity of the different methodologies, enabling automated sizing of fenestrated SG [27].

#### 2.3.1. Applications in Abdominal Aortic Aneurysms

- Proximal sealing zone prediction.

Endovascular abdominal aortic aneurysm repair (EVAR) success depends mainly on the appropriate SG selection and sizing, especially in complex vessel morphologies, such as hostile neck and arterial tortuosity. Numerical prediction of proper EVAR deployment in a particular anatomy has the potential to significantly reduce peri-operative and long-term complications.

In 2014, De Bock et al., were the first to describe a virtual model of SG deployment in an angulated proximal neck with various degrees of angulation, length, and diameters using FE analysis [26]. They showed that the most adverse effects were obtained with high angulations and short, straight landing zones. However, these results were described in virtual anatomies, which limits their proper validation. Hemmler et al., developed an EVAR placement model with a direct placement method, considering both SG and aortic models as elastic deformable bodies. They demonstrated its applicability with patient-specific cases and SG and AAA parameter studies [15,38,40]. Their most recent study focused on challenging aortic necks and tested the impact of SG customization on proximal fixation in idealized proximal neck anatomies [28]. This study focused on aortic neck geometry and did not take into account the potential impact of aortic neck calcification or thrombus. Various parameters were tested (number, positions, types of stent-rings, and morphology of SG adjusted to vessel morphology, such as curvature, conicity, etc.). SGs were standardized with polytetrafluoroethylene (ePTFE) material for the graft and sinusoidally shaped nitinol stent-rings of constant height and wire thickness, without adaptation, for different manufacturers’ designs. Interestingly, they found benefits of customized SGs with an increase in SG fixation forces of up to 50% and the authors suggested the development of highly customized SGs for use in challenging anatomies. Validation in patient-specific anatomies should be conducted to confirm these promising results.

To improve the EVAR proximal seal, endo-anchors were added for short proximal necks. Recently, Abbott et al., analyzed the impact of endo-anchor number and position on SG apposition in one patient’s abdominal aortic aneurysm using FE analysis [41]. They found that endo-anchors improved fixation within the proximal seal zone and under biomechanical conditions of high peak systolic pressure when compared with no endo-anchors or medial and distal endo-anchor positioning. Although this study was limited by a single patient computational design and model simplifications, the feasibility of endo-anchor simulation was demonstrated and its clinical improvement on sealing was numerically confirmed.

- Arterial deformation by device introduction and its impact on sizing

During EVAR, iliac tortuosity can lead to unintentional coverage of the intern iliac artery. Therefore, a team focused on arterial deformation induced by stiff guidewires and delivery system introduction in iliac arteries and aorta [39,42,43]. Their most recent work analyzed deformations in two groups (*n* = 38), showing retrospective prediction of accidental iliac intern coverage with a sensitivity of 100% and specificity of 60.6%. Model improvements should help obtain a predictive value that would allow prospective validation and its use in clinical practice.

#### 2.3.2. Applications in Thoracic Aortic Aneurysms

In the past decades, thoracic endovascular aortic repair (TEVAR) has been widely disseminated, with high impact on reducing morbidity and mortality compared to open surgery. However, issues remain, such as endovascular treatment of the aortic arch or ascending aorta, with dramatic consequences of accidental coverage of collaterals in this area.

- Endovascular treatment for the ascending aorta

Endovascular treatment of the ascending aorta is not recommended and currently reserved for compassionate cases [44]. Indeed, high constraints and lack of dedicated devices make this technique unsafe and uncertain. To help practitioners, Auricchio et al., were the first to report FE results on the deployment of a tubular SG in the ascending thoracic aorta and its model was used few years later for 3 other patient-specific cases, suggesting that simulations can help to choose the right SG for the ascending aorta, as well as the right deployment sequence [14,45]. Arokiaraj et al., described a numerical model for bare stent deployment in virtual anatomy of an ascending aorta aneurysm and analysis of its impact on aortic remodeling. They showed that the wall shear stress was reduced [46]. The main limitation of these studies is the assumption regarding the arterial wall. Considering the vessel wall as a rigid surface simplifies the model, which could be reasonable for specific purposes and conditions. However, it may be a strong approximation for complex problems, especially in the ascending aorta, where anatomical and hemodynamic constraints are high.

- Aortic arch endovascular treatment planification

Total endovascular repair of the aortic arch is a highly complex procedure, in which the results are related to operator experience [4]. Derycke et al., first described the complete simulation of deployment of a complex Terumo Aortic^®^ Relay Branch device and its bridging stents in an aneurysmal aortic arch [47]. The complex design of the branched SG implied a long calculation time but its potential impact on clinical practice is high, considering that the aortic arch remains one of the last challenges of total endovascular aortic repair with major complication risks. A focus on this issue is provided in the next chapter.

- Descending aorta endovascular treatment planification

Le Berre et al., described the simulation deployment of stents in a thoracic aortic aneurysm with angulated anatomy, reporting the impact of neck length and oversizing on proximal migration risk [48,49]. Although interesting, the main limitations of this work were the lack of validation and absence of graft modeling. Indeed, part of the stents’ radial force due to their oversizing compared to the graft diameter was neglected, which can greatly impact proximal sealing.

Caimi et al., recently described SG deployment in aortic coarctation for three patient-specific cases and analyzed the stent and aortic stresses [50]. Further validations should be conducted, but this approach is promising for supporting patient and device selection.

Recently, results were reported in virtual anatomy. First, Ramella et al., reported results of SG deployment in descending aorta in an idealized anatomy. They validated their results using a 3D-rigid aortic phantom model [51]. Then, Shahbazian et al., focused on the bird-beak configuration after TEVAR and analyzed thoracic SG deployment in different virtual anatomies. Different values of aortic curvature, arch angle, landing zone, SG oversizing, and aortic tissue properties were tested. The results highlighted an impact of these parameters on the bird-beak configuration [52,53]. However, proper validation in real anatomies must be achieved to confirm these promising results.

#### 2.3.3. Applications in Thoraco-Abdominal and Juxta-Renal Aortic Aneurysms

- Arterial deformation by device introduction and its impact on sizing

A crucial step for technical success of endovascular repair of the thoraco-abdominal aorta is the accurate positioning of the branches and fenestrations.

Device introduction in case of arterial tortuosity can lead to SG torsion during deployment, which could be predicted by simulation. Indeed, torsion following guidewire insertion was simulated by Sanford et al. [54]. Their model was able to predict the torsion effect of a fenestrated SG for six patient-specific models. Then, Dupont et al., published results on fenestrated SG deployment with validation on one patient-specific case with superimposition of the real stents extracted from the post-operative CT scan and the simulated stents. Good alignment was obtained at the level of the visceral aorta. This work was based on previous studies that validated a model able to predict aorto-iliac deformations induced by stiff guidewires and delivery system insertion [39,43]. After determining the optimal simulation parameters for the first case, they studied the impact of stiff guidewire insertion, delivery system insertion, and SG delivery on the position of visceral ostia [55]. Significant displacement of the renal arteries was found, which could impact per-operative catheterization difficulties.

- Procedural planification

Derycke et al., conducted retrospective simulation of 51 patient-specific cases of Zenith^®^ fenestrated devices (Cook medical), showing good accuracy for the sizing of target arteries obtained by simulation when compared with manufacturer sizing and post-operative CT scan [27]. Based on the same FE analysis model, Kliewer et al., recently published the results of a prospective study in six European centers. 50 patients treated using the Fenestrated Anaconda^®^ (Terumo Aortic) were compared for sizing based on 3D in vitro models and simulation, showing satisfactory accuracy [56]. A significant decrease in delivery time can be obtained with simulation planification. As a result of this study, simulation results are now used by the manufacturer in the design process of Fenestrated Anaconda^®^ devices.

#### 2.3.4. Applications in Aortic Dissections

- Retrograde type A dissection

Retrograde type A dissection post TEVAR is a major and potentially fatal issue. Indirect risk factors were identified, such as connective tissue diseases, ascending aorta diameter, or bicuspid aortic valve. WSS caused by TEVAR could be analyzed with FE analysis to provide additional and more personalized information. The effect of oversizing and connecting bar SG was assessed in 2018 and 2020 by one research team for Medtronic^®^ devices [57,58]. Numerical deployment simulations were achieved in two patient-specific cases of acute type B aortic dissections. The results showed that maximum stress occurred at the junction of the bare stent with the greater curvature of the aorta. Moreover, the stress was significantly higher in the case of the connecting bar and increased linearly with oversizing. Complementary work focusing on retrograde dissection cases could be more informative in determining the level of WSS associated with retrograde dissection. Moreover, current practice recommends the use of non-bare SG in acute type B aortic dissection. Simulations of such devices could be even more relevant.

- Aortic wall remodeling

Recently, the same team evaluated stress distribution in two different SG length configurations with an improved model [59]. The new model took into consideration pre-stress of the aorta, hyperelastic properties, and separate material properties for the intimal flap. The length of the aortic curvature remains an issue in acute type B aortic dissection, as a balance must be reached between achieving sufficient aortic remodeling and reducing the risk of spinal cord ischemia. In this study, the authors analyzed the distribution of Von Mises stress on the intimal flap and outer wall. The Von Mises stress value is used in FE analyses to determine if a given material will yield or fracture. The authors showed that the Von Mises stress was reduced with longer SG in one particular case and suggested that its use might have prevented the development of a distal new entry that was observed at the 3-month follow-up. Multiple cases will be needed to obtain an in-depth understanding of the impact of Von Mises Stress on new entries.

- Endovascular treatment of type A dissection

Endovascular repair represents a future solution to treat type A aortic dissection. As for aneurysm diseases, due to the lack of a dedicated SG design and the complexity of its deployment in the ascending aorta, indications are reserved for non-operable cases with favorable anatomy. Numerical simulation has shown potential in predicting outcomes and planning future endovascular procedures [60]. A Gore^®^ CTAG was numerically deployed in an ascending aorta under two scenarios: the first one reproduced the actual procedure and migration of the SG in the false lumen, the second deployed the 10 mm SG proximally, obtaining better results with the absence of migration and a decrease in the wall shear stress at the level of the dissection lamella and entry tear. Further validation is needed to confirm these promising results.

## 3. Focus on Two Specific Clinical Applications

With applications in complex endovascular aortic repair, numerical simulation may be a new tool that challenges how we image, plan, and carry out cardiovascular interventions. Two specific clinical applications are presented in the following chapter as concrete examples.

The methodology was developed by Demanget et al., and then, Perrin et al., [5,25,32,61]. They reported the results of iliac extension and EVAR simulation with a virtual shell method, explained later, and validated the results in standard and tortuous patient-specific anatomies with various device designs.

### 3.1. Planification of FEVAR

Fenestrated SG has become a standard endovascular approach to treat complex abdominal aortic aneurysms. The devices are custom-made and specifically tailored to each patient’s anatomy. Actual planification is based on manual measurements of different anatomical characteristics on a dedicated workstation, which can be challenging in complex arterial anatomies. Poor anticipation of SG deployment can lead to severe intra-operative difficulties. An automated, reproducible, and precisely sized procedure could facilitate and secure the clinical process. Numerical simulation with FE analysis was assessed for this issue [27].

The work was based on proprietary algorithms (PrediSurge, Saint-Etienne, France) developed to simulate the deployment of aortic devices in patient-specific abdominal aortic aneurysm models [25,32]. The patient-specific aortic models and corresponding FEVAR devices were numerically reproduced for 51 patients who underwent implantation of a Zenith^®^ fenestrated AAA endovascular graft (Cook Medical, Bloomington, IN, USA). the FEVAR deployment methodology followed the virtual shell method: the aortic surface was virtually deformed until a cylindrical shape was obtained. The SG was then deployed in the cylindrical aorta and maintained inside while the aortic surface was deformed again to its original shape.

**Figure 2:**
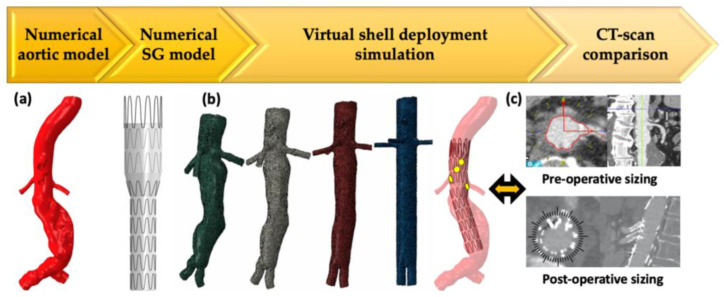
Methodology of FEVAR simulation. (**a**) Aortic and stent-graft modeling. (**b**) Virtual shell step and results of deployment simulation with fenestration positioning. (**c**) Results are compared with standard planning technique and post-operative computed tomography scan measurements. SG: stent-graft, CT: computed tomography.

The position of each target artery on the SG was measured in the simulation results, manually, and using an automated algorithm. Data were compared with those obtained from the pre-operative CT scan by the planning center and on the post-operative CT scan by an independent senior surgeon. The different steps are illustrated in Figure 2.

The statistical analyses showed good reproducibility between the three methods of measurement. A total of 95% of the measures were under 3 mm for longitudinal deviance and 15° for rotational deviance. Moreover, the simulations allowed qualitative assessment with two cases of proximal suboptimal apposition, one case with an excess of oversizing, six cases of inhomogeneous deployment, and six cases with pleated fabric at the level of fenestrations. Superimposable aspects were present on the post-operative CT scan.

This study showed that numerical simulation of FEVAR was feasible and accurately determined patient-specific fenestrated positions when compared with the gold standard method. The clinical impact is high considering the complexity of planification and its actual uncertainty. Intra-operative difficulties could be avoided with pre-operative numerical simulation, particularly in challenging anatomies.

### 3.2. Outcome Prediction in the Aortic Arch

Total endovascular repair of the aortic arch is a complex procedure associated with numerous challenges, including device design, alignment, and durability. Numerical simulation, based on SG and patient-specific aneurysm models, may help to predict SG behavior during deployment inside the aorta.

Derycke et al., extended the approach to simulate complete deployment of a complex Terumo Aortic^®^ Relay Branch device and its bridging stents in cases of aneurysmal aortic arch [47,62]. This work was based on the same proprietary algorithms (PrediSurge, Saint-Etienne, France) and previously described methodology.

**Figure 3:**
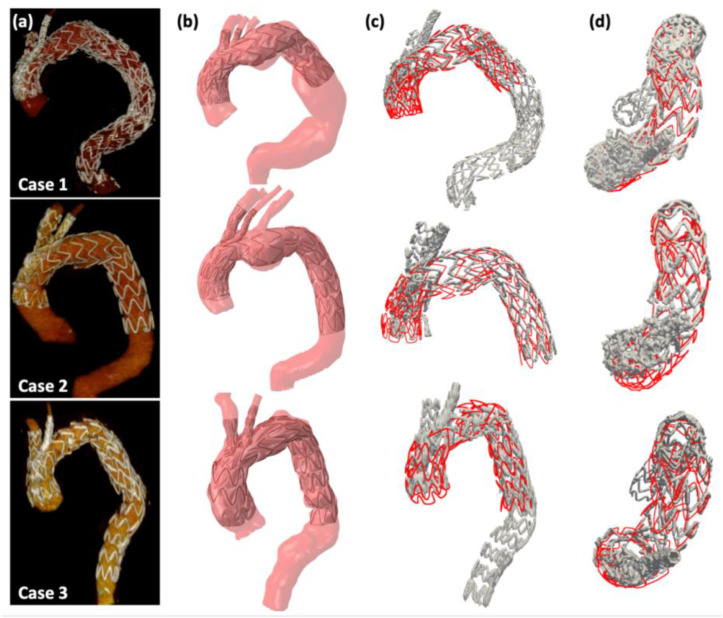
(**a**) 3-dimensional reconstructions of post-operative CT scans for the three cases. (**b**) Simulation results. (**c**) Sagittal views of the qualitative comparison between stent-rings extracted from simulations (in red) and post-operative CT scan (in grey). (**d**) Transversal views.

Patient-specific numerical simulations of three patients were evaluated. Quantitative and qualitative comparisons of 3-dimensional stent shapes between numerical models and post-operative scans were performed, showing satisfactory accuracy, as shown in Figure 3.

**Figure 4:**
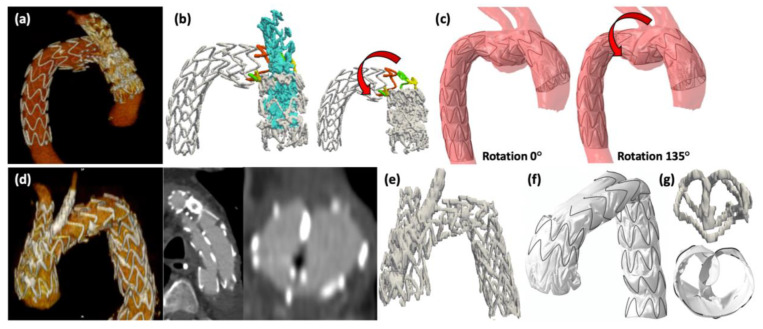
(**a**) 3-dimensional reconstruction of post-operative CT scan of case 2. (**b**) Stent-rings extracted from the post-operative CT scan: the half stent-rings of the fenestration zone are colored in yellow, green, and red and the bridging stents are colored in blue. The arrow indicates the torsion effect. (**c**) Simulation results with 0° and 135° rotation: torsion appearance and correct length are obtained with 135° rotation. (**d**)**.** 3-dimensional reconstruction of post-operative CT scan of case 3 and post-operative images showing the wrong expansion of the three stents located at the aortic isthmus level with infolding. (**e**) Stent-rings extracted from post-operative CT scan. (**f**) Coronal view of the simulation result. The arterial surface was omitted from the overview to facilitate visualization. (**g**) Cross-sectional views of the stent-rings extracted from post-operative CT scan (on top) and of the simulation result (at bottom) at the aortic isthmus level showing infolding.

**Figure 5:**
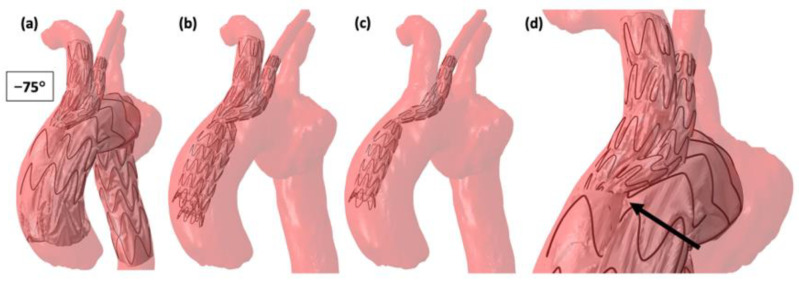
(**a**) Simulation result of deployment with −75° rotation. (**b**) Picture of simulation result without the main stent-graft to facilitate visualization. (**c**) Picture showing only the bridging stent corresponding to the left carotid artery. (**d**) Simulation result: the arrow indicates the incongruence.

Inadequate deployment, SG torsion with significant shortening of the SG in one case, and major SG infolding in another, were successfully reproduced by simulations (Figure 4). The impact of torsion related to advancement of the delivery system through tortuous iliac arteries on the bridging stent configuration was also evaluated, showing that a large degree of torsion was allowed without consequences on the bridging stents. Indeed, a kink was only observed on bridging stents at −75° of rotation, as shown in Figure 5.

These studies showed that numerical simulation may be used to predict deployment of complex devices in the aortic arch. This technique offers several potential advantages over currently used methods of pre-operative planning. Design and dimensions can be validated by direct visualization of the different SG components deployed in a personalized aortic model. Adequate application of the main device onto the aortic wall proximal and distal to the supra-aortic trunks may be verified. Moreover, inadequate deployment, such as infolding related to excessive SG oversizing or branch kinks due to inappropriate rotation of the main aortic body, may be anticipated. Such personalization of pre-operative planning may improve patient selection, allowing testing different SG designs and intervention strategies. This may result in a dramatic reduction in intra-operative technical difficulties and, therefore, post-operative mortality and morbidity. However, assistance of FE simulations for total endovascular repair remains challenging as it requires sophisticated numerical models that still need to be validated on a sufficient number of patients.

## 4. Current Challenges of FE Analysis in Endovascular Surgery

The aim of computational analysis is to obtain good accuracy of the model, but approximations are needed to define algorithms compatible with computer capacities and reasonable calculation times. Therefore, FE analysis results must be interpreted with caution, especially when the approximations are strong and the results are not validated by a clinical or in vitro model.

Several different models of SG placement were developed to predict final SG deployment in the aorta, rather than reproducing all single steps of the real interventional procedure. Indeed, precisely replicating the process is numerically challenging, time-consuming, and leads to numerical instabilities that would cause algorithm failure. Three methods are currently used, including the virtual catheter method, virtual shell method, and direct placement method, and have been validated on a small cohort basis [14,25,38]. The virtual catheter method was used in simulation models to radially crimp the SG by a cylindrical delivery catheter, bend and position the SG onto the aortic centerline, and then, deploy the SG by enlarging the diameter of the catheter [14,33,45]. The virtual shell method was developed by Perrin et al., and uses a morphing algorithm [25,32]. The aortic surface is deformed until an idealized cylindrical shape is obtained. Then, the SG is deployed and the aortic wall is deformed again into its original geometry. Finally, the material properties of the aorta are applied in order to deform the aorta in response to forces exerted by the SG. The direct placement method was developed by Hemmler et al., and consists of direct application of displacement constraints to the SG using a 3D morphing algorithm based on SG and AAA centerlines [15,38]. A similar concept was used by Dupont et al., with a preliminary step of deforming the aorta and iliac arteries by stiff guidewire insertion [55].

The application of the virtual catheter method appears to be limited to non-bifurcated SGs, as multiple virtual catheters are required for bifurcated devices, which significantly increases model complexity. The virtual shell method is more computationally efficient than the virtual catheter method, since it allows modeling simplification [25]. However, in case of tortuous and mobile arteries, a significant loss of accuracy can be observed and additional steps are required to obtain realistic results. The direct placement method reduces computational complexity, allowing assignment of more complex material properties [38]. Finally, the choice of the method depends mainly on the experience of the research team, the endovascular procedure that is simulated, and the FE software used.

Material properties are also subject to approximation. The aortic tissue is composed of three layers with varying characteristics. In the presence of an aneurysm, calcification, or intra-luminal thrombus, these characteristics change locally and globally [8,11,63,64,65,66]. Moreover, mechanical properties depend on the level of the aorta (thoracic, visceral, abdominal) [9]. When modeling the aortic wall, all of these factors need to be accounted for. Hyperelastic models of anisotropic fibrous composites (collagen fibers embedded into a ground matrix) can be considered as the most accurate material property definition. In FE analysis and endovascular modeling, such precise definition is rarely used, and orthotropic elastic or even rigid model have been previously used.

SGs are composed of stent-rings and fabric. Common materials of commercial SG devices are stainless steel or nitinol for stent-rings and polyethylene terephthalate (PET or polytetrafluoroethylene (ePTFE) for the graft. After tests with homogenous SG models, which were inaccurate, more elaborated SG models were developed. The anisotropic material property is frequently approximated by isotropic elastic models for the fabric and orthotropic elastic or super-elastic models for stent modeling. These approaches have been validated in small cohort studies [5,7].

FE analysis models neglect the interactions between blood and the device. Major possible clinical complications, such as endoleak and graft thrombosis, are post-operative and related to these interactions with blood. Their prediction would require sophisticated models involving multiphysics. The implementation of fluid-structure interactions to assess blood flow and therefore predict such effects has rarely been achieved [67] and this research direction deserves important efforts in the future.

Computation times are still relatively long, mostly due to the large number of degrees of freedom and necessity to solve a large number of contacts. One of the most urgent challenges in the field is to use reduced order models. Reduced order modeling is a family of methods that aim at reducing the computational complexity of numerical problems by approximating their solution. In practice, these techniques, such as reduced basis (RB) methods, replace the high-fidelity, full-order model (FOM) with a reduced order model (ROM) characterized by a much smaller dimension. RB methods have successfully been used for cardiovascular applications [68], such as arterial blood flow and heart electrophysiology modeling, but extension to the simulation of endovascular procedures remains a work in progress [69,70].

## 5. Future Directions

FE analysis could be used as a sizing and predictive tool for aortic endovascular intervention. Its rigorous validation for simple aortic anatomies could then extend its scope to complex anatomies. Moreover, it could not only help in planification, but even assist practitioners during the procedure. In this way, the creation of a worldwide data registry could help to refine and verify results by providing a large volume of patient data. Numerical simulation could enable uncertainties to be quantified with greater accuracy, thus defining the degree of accuracy to be expected for each individual case. Indeed, the results depend on various parameters, such as arterial anatomy, age, presence of calcifications or aortic wall thrombus, and arterial tortuosity.

Similar to an airplane or car being numerically tested by the industry, short- and long-term fatigue behavior of endovascular devices could also be assessed under cyclic stress between diastolic and systolic phases for fatigue stress analysis of the SG material [71]. Alternative designs could be investigated without additional manufacturing costs.

Aortic dissection disease is a separate entity, which involves hemodynamic and geometric parameters and location of tear(s) that can be hard to measure and understand in vivo. Consequently, its natural history is difficult to predict and its best management is controversial. Numerical simulation could provide answers. A recent review analyzed 124 patient-specific models in 37 studies, with a large majority being CFD studies [72]. Indeed, CFD allows wall shear stress analysis and measurements of false and true lumen pressure and flow velocity that could help to predict thrombosis, false lumen flow, development of retrograde dissection, or malperfusion syndrome. Significant concordance was found between studies, despite a relatively limited number of patients and heterogeneity in the methodologies. The current challenge is to develop a risk assessment method for complicated type B dissection that can be used in clinical practice. The use of numerical simulation of TEVAR deployment with FE analysis, as a complement to CFD analysis, could provide another perspective, as different parameters could be assessed before and after TEVAR in a particular anatomy.

The concomitant use of FE analysis and ML frameworks has the potential to greatly facilitate some steps and save computation time. Nevertheless, ML still need to be improved before being implemented in routine clinical practice. The selection of ML algorithms should be made based on the questions of interest and the structure of the dataset (population, number of cases, time to event, type of outcome, etc.) [73]. There is, therefore, a need to standardize ML methodologies and perform validations in large cohorts to confirm their application to sample data [74,75].

Similarly, there is a pressing need for thorough and extensive verification, validation, and uncertainty quantification of FE analysis for endovascular procedures, as was previously achieved in other domains of biomedicine [76]. The clinical impact of numerical simulation, which has already been assessed retrospectively, could be assessed by prospective randomized trials.

Hemodynamic factors, induced by pulsatile blood flow, play an important role in vascular pathologies, especially in atherogenesis [29]. For endovascular procedures in complex aortic pathologies, computational fluid dynamics have been extensively used to provide information that could not be obtained from standard vascular images. The wall shear stress, which quantifies the viscous shear applied on the endothelial cells due to blood flow, has been extensively studied [29]. CFD is particularly relevant in aortic dissections, as it could play a role in the decision-making process [72]. Indeed, it remains difficult to identify patients who initially present uncomplicated type B aortic dissection but will develop mid- or long-term complications. Analysis of entry or exit tears and false lumen flow rates could provide additional information, as well as help develop risk prediction models [72]. Although insightful data have been found, the heterogeneity and relative number of patients in the current literature limit actual application. Other applications include endoleaks and their consequence on pressure in aneurysm sacs and hemodynamics in target vessels, such as visceral arteries in FEVAR and chimney in EVAR, which may help better understand intra-stent stenosis or thrombosis [77]. Finally, as for structural mechanics, model results depend mainly on the quality of the information used to build the computational models. Current models are often simplified since completely personalized simulations are highly challenging [72].

## 6. Conclusions

Endovascular management of complex aortic diseases involves various issues that could be explore by FE analysis. Currently, few studies have shown sufficient validation to be used in routine clinical practice and the technology is still in its infancy for the most complex SG devices. Nevertheless, for a number of challenges, numerical models are not far from being ready to provide solutions and help practitioners choose the most tailored and straightforward solution for each patient.

## Data Availability

Not applicable.
